# A catastrophic presentation of dead limb secondary to traditional bone setter's treatment in Sorong, West Papua: A case report

**DOI:** 10.1016/j.ijscr.2021.106061

**Published:** 2021-06-11

**Authors:** Lu Jordy Luhur, I Putu Gede Dharmawan, I Gusti Lanang Ngurah Agung Artha Wiguna

**Affiliations:** a Emergency Department, John Piet Wanane General Hospital, Sorong, Indonesia; b Department of Orthopaedic and Traumatology, John Piet Wanane General Hospital, Medical Faculty of Papua University, Sorong, Indonesia; c Department of Orthopaedic and Traumatology, Sanglah General Hospital, Medical Faculty of Udayana University, Bali, Indonesia

**Keywords:** Gangrene, Extremity, Bone setter, Amputation, Traditional treatment, Case report

## Abstract

**Introduction:**

Traditional bone setter's gangrene is a devastating complication arising from the practice of TBS with a prevalence of 4.29%, and most cases require amputation, which led to permanent disability. This case demonstrates a catastrophic presentation of the lower limb gangrene secondary to traditional bone setter's treatment.

**Case presentation:**

A 39-year-old man with history of treatment by traditional bone setter presented with generalized pain with loss of the skin throughout the entire right lower leg, leaving soft and hard tissue exposed to the environment. His vital signs showed tachycardia and fever. Pulses were absent on the entire lower leg except for the femoral artery. The radiograph revealed gas density around the soft tissue suggestive of gas gangrene. After stabilization, an above-knee amputation was performed by the orthopedic surgeon.

**Discussion:**

Traditional bone setter's gangrene is a significant contributor to amputations in many developing countries. The practice of scarification, massage with herbal concoctions and creams, and a tight splint may lead to infection, vascular compromise, compartment syndrome that may terminate in gangrene or death of the limb. This progressive limb-threatening infection emphasizing the importance of early recognition of compartment syndrome, adequate resuscitation or stabilization, and prompt and aggressive treatment.

**Conclusion:**

This case highlights the menace of TBS activities and the dangers inherent in their practice. People who are misguided by false beliefs should be educated by public enlightenment. Appropriate legislation should be supplemented by the government to integrate traditional bone settings with the new orthopedic care services.

## Highlights

• Traditional bone setter’s practice has been associated with unacceptable outcomes such as gangrene
• This practice lacks the fundamental scientific principles of fracture management and infection prevention and control
• Limb gangrene eventually can lead to sepsis. Proper medical and surgical intervention are necessary
• This case presents a potential benefit of amputation in a patient with limb gangrene and progressive deterioration

## 1 Introduction

Traditional bone setter's (TBS) practice has been associated with unacceptable outcomes in many musculoskeletal cases. Complications arising from their practice significantly contribute to the challenges facing the orthopedic practitioner. These complications include malunion and nonunion from lack of radiographic imaging or proper reduction, compartment syndrome and gangrene from constrictive immobilization, and infection from lack of sterility, prophylaxis, and scarification [1]. People in developing countries are still looking for traditional healers to solve their health problems, including fractures. Most fracture cases continue to be treated by TBS, readily available, and have a good reputation among the locals. Treatment by TBS is a very specialized method of traditional medicine that is usually preserved along family lines with limited opportunities for non-family members to learn the practice via apprenticeship [2,3].

In Papua Province, TBS is an age-long practice and existed long before the arrival of orthodox medicine, making the TBS enjoy high patronage from the community. There are 17.18% who choose TBS as their choice of treatment, and 81.67% using self-made concoctions [4]. Despite limitations in traditional practices, some demands or patient-related factors, such as ignorance, peers and family pressure, poor socioeconomic status, aversion for implants, fear of amputation, cultural beliefs, and affection of concoctions and incantations, contribute to the support of traditional medicine [3].

The practice of bone setting is unregulated and lacks the fundamental scientific principles of fracture management as well as infection prevention and control. The technique mainly applies herbal and earthen mixtures to the limbs and then improperly fixes them with wooden splints without resorting to anatomy, physiology, or radiology. Frequently, the splint is tight, which can lead to compartment syndrome and gangrene or death of the limbs. Bone setter's gangrene occurs with a prevalence of 6.6%, and it is the most catastrophic of all complications arising from fracture and non-fracture treatment by the TBS. The only treatment for gangrene is amputation, and this is devastating to the patient and family members, even when it is the only life-saving option. The loss of the limb results in a lifetime disability and stigma, impacting the patient and the family [[5], [6], [7]].

With the patronage enjoyed by the TBS in many low- and middle-income countries, complications of fracture care ranging from limb- to life-threatening conditions have persisted and remained a significant challenge to the orthopedic surgeons practicing in these regions. We reported a patient who refused treatment from the hospital and attended to TBS, which led to severe progressive limb-threatening infection. This case report has been reported in line with SCARE criteria [8].

## 2 Case presentation

**Fig. 1:**
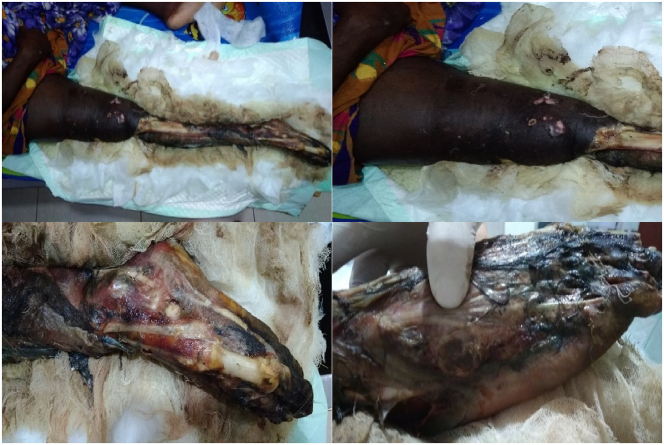
Clinical view of the right leg showing the area of gangrene.

A 39-year-old male farmer was referred to our emergency department with generalized pain on the right leg with loss of the skin throughout the lower leg leaving the soft and hard tissue exposed to the environment shown in Fig. 1. He could not move his right leg and feel no sensation at some part of the limb. No history of loss of consciousness, amnesia, and headache was reported. He was a smoker and occasional drinker but did not have allergic and vascular risk factors such as hypertension, diabetes, or dyslipidemia. There was no history of medication use.

Two weeks earlier, he had a motorcycle accident and crashed into a truck with the right leg as the focus of impact. He was immediately brought to the hospital, where he was taken plain X-Ray on the right leg with suspicion of open fracture of the right lower limb. Clinically, the patient was alert and complained that he had intense pain and unable to move the right leg. The doctor explained the urgent need for operation as there was an open fracture observed, but the patient decided to discharge against medical advice and sought home treatment with a TBS. During the treatment, the leg was directly manipulated by holding the patient down, while the TBS performed the manipulation without using analgesics. The traditional herbs were used in the mixture and blended into a cream that was applied to the lower leg right after the manipulation. Afterward, the splint was applied over the herbal cream, and a bandage was put around tightly to hold the splint in place.

After 24 h, he started to feel intense pain, swollen and numbness throughout the right leg. He had a fever that developed in the following days with limb exuding foul-smelling discharge. Progressive darkening of the limb was noticed, and the leg was swollen. He was subsequently brought to the hospital and treated for five days. Before referred to our hospital, his vital signs were reported as blood pressure 90/60 mm Hg, pulse rate 110 beats/min, respiration rate 20 breaths/min, temperature 38.3 °C. Laboratory results in Table 1 indicated severe anemia (hemoglobin was 4.6 g/dl) and severe infection (white blood cell count was 29.3×10^9^/l). He was given intravenous fluid, analgesics, antipyretic, broad-spectrum antibiotics, and blood transfusion.

**Table 1:** Laboratory results.

Laboratory parameters	Values				
	Before stabilization	After stabilization	1st day of admission	3rd day of admission	4th day of admission
Hemoglobin (g/dl)	4.6	8.7	8.6	12.2	11.2
Hematocrit (%)	13.3	26.9	26.5	36.4	32.9
White blood cell count (×10^9^/l)	29.3	12.9	9.7	9	8.1
Platelet (×10^9^/l)	443	330	273	299	273
Erythrocyte (×10^6^/l)	1.71	3.19	3.36	4.67	4.21
MCV (fL)	77.9	84.4	78.9	77.9	78.1
MCH (pg/cell)	26.8	27.2	25.6	26.1	26.6
MCHC (g/dl)	34.4	32.2	32.5	33.5	34.0
Differential counts					
Basophils (%)	0	1	0.8	0.5	0.3
Eosinophils (%)	0	3	1	1.5	2
Neutrophils (%)	90	89	90.7	89.7	87
Lymphocytes (%)	3	5	2.8	4.9	9.1
Monocytes (%)	7	2	4	5.4	6
SGOT (u/l)	14	–	27	–	–
SGPT (u/l)	20	–	28	–	–
Urea (mmol/l)	38	–	34	–	–
Creatinine (mmol/l)	0.8	–	0.6	–	–
Albumin (g/l)	4	–	4	–	–
Random blood glucose (mg/dl)	119	–	160	–	–
HbsAg	–	–	Negative	–	–
HIV	–	–	Negative	–	–
PT (second)	–	–	10.3	–	–
APTT (second)	–	–	35.3	–	–

**Fig. 2:**
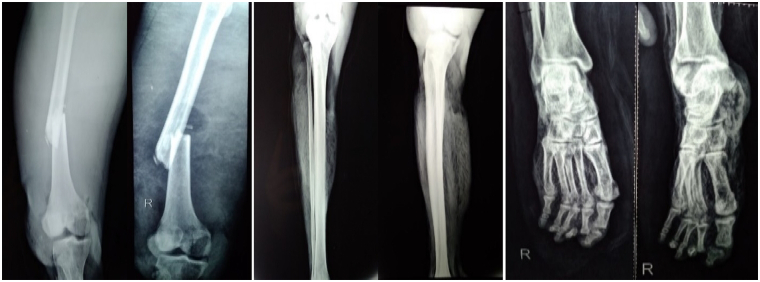
Anteroposterior (AP) and lateral (L) X-Ray view of the right lower extremity. Left: AP/L X-Ray view of the upper leg. Middle: AP/L X-Ray view of the lower leg. Right: AP/Oblique X-Ray view of the foot.

Physical examination at presentation showed he was alert with vital signs showing a blood pressure of 100/60 mm Hg, a pulse rate at 96 beats/min, respiration rate at 20 breaths/min, a temperature of 37.8 °C. The Visual Analogue Scale (VAS) was 7. The results of the general examination were within normal limits. Essential findings in the musculoskeletal system of the right leg were the loss of skin tissues from the knee to the foot. The bone and the soft tissues such as muscles and tendons were exposed, dirty, darkened, and dead, leaving a foul smell. The knee was held in a fully extended position. Palpation of the limb showed crepitation at the level of the femur, and the temperature of the gangrenous part was cold. Range of motion (ROM) examination showed there was no active movement below the knee joint. Normal pulse was palpated at the femoral artery, but there was no pulse at dorsalis pedis, posterior tibial, and popliteal artery. No sensation felt throughout the lower leg and no active movement below the knee joint. His laboratories at the time of admission showed: hemoglobin 8.6 g/dl, hematocrit 26.5%, erythrocyte 3.36×10^6^/mm^3^, white blood cells 9700/μL (diff. Count of neutrophils 90.7%, lymphocyte 2.8%). All other laboratory values were within normal limits, shown in Table 1. Anteroposterior (AP) and lateral (L) view X-Ray of the upper leg showed displaced transverse fracture of the middle third of the femur. AP/L view of the lower leg showed an incomplete fracture of the middle third of the fibula, soft tissue defect at the proximal third and distal third of the tibia, and gas density around soft tissue suggestive of gas gangrene. AP/oblique view of the foot showed an oblique fracture of the 5th metatarsal, dislocation of the middle phalanx of the 2nd digit, subluxation of the distal phalanx of the 4th digit, and gas density around the soft tissue suggestive for gas gangrene. Fig. 2. Biopsy could not be performed due to financial reason.

**Fig. 3:**
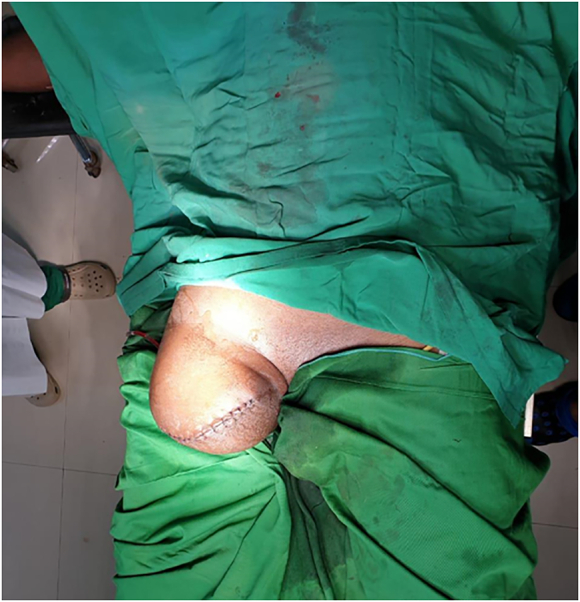
Postoperative clinical view of the amputated right leg at the level of femur (above-knee amputation).

With the above physical and imaging characteristics, a diagnosis of dead limb secondary to bone setter's treatment was made. The patient and his family were informed about the urgent need for amputation after stabilizing the patient's condition. He was given intravenous fluid, antibiotics (ceftriaxone, 1 g every 12 h; gentamycin 80 mg every 8 h), analgesics (ketorolac, 30 mg every 8 h), antipyretics (paracetamol, 1 g every 8 h IV drips), and blood transfusion one bag every 24 h until the hemoglobin reaches the minimum of 10 g/dl. He underwent above-knee amputation by an orthopedic surgeon in our center on the 3rd day of admission Fig. 3. This was considered as the limb was non-functional, the inconvenience of wound care, and the risk of becoming a focus of infection.

Postoperative recovery was excellent, surgical drain and the urethral catheter were removed after 72 h, and on the 5th postoperative day, he was discharged with suggestions for routine wound control in the orthopedic outpatient department. He was given oral antibiotics (cefixime 200 mg every 12 h, metronidazole 500 mg every 8 h), analgesics (mefenamic acid 500 mg every 8 h), ferrous sulfate 200 mg every 12 h, and calcium lactate 500 mg every 24 h. One week follow-up of the patients showed satisfying wound control, and he was referred to rehabilitation for exercises such as; standing, balancing, and endurance exercise. After two weeks, he presented without any additional complaints and was grateful for his condition.

## 3 Discussion

TBS's gangrene is a widely reported complication with a prevalence of 6.6%, and 63.6% involved the lower limb [9]. TBS's gangrene is a significant contributor to amputations in many developing countries. The resulting loss of the limb is unwarranted and is both an avoidable and preventable disaster. Limb loss can result in significant morbidity, disability, and profound economic, social and psychological effects to the patient and family, especially in developing countries where prosthetic services are inadequate [10]. Chika et al. reported that 15% of amputations in Nigeria were due to bone setter's gangrene, while nearly a quarter of all trauma-related gangrene were bone setter's gangrene [6].

The reason behind patronizing TBS in this study was advice from the family and relatives. The influence of relatives is important because of the existing social system in Papua, where family and friends will normally contribute to treatment choice. A study in Cameroon showed that the advice of relatives and friends was the most common reason for TBS patronage, which constitutes about 30.6% of patients. Other reasons were cheaper cost (24.5%), sociocultural belief (14.3%), easy accessibility (12.4%), fear of amputation (10.2%), and fear of operation (8.2%) [11]. However, Dada et al. reported that perceived cheapness (27.9%) becomes the major concern for patronizing TBS, followed by pressure from family and friends (25%) [7].

The TBS's practice of scarification, massage with herbal concoctions and creams, and tight splint to prevent fracture movement may lead to infection, vascular compromise, compartment syndrome, ischemia of muscles, and nerves with pressure on the skin and bony prominences. That practice sets up a cascade resulting in complications that may terminate in gangrene or death of the limb [9]. Early studies stated that irreversible nerve and muscle damage begins after 5 to 6 h of ischemia. More recent clinical studies revealed that muscle necrosis occurs within the first 3 h. Muscle tissue reacts to ischemia with scar formation resulting in adhesions and contractures [12].

This case demonstrated the presence of the dead limb with suggestive of pre-existing compartment syndrome characterized by the history of intense pain, which is usually out of proportion to the degree of injury after the splints are applied. Unfortunately, the pain was unrecognizable, as it was taken as a normal response to the injury. The tightness of the splint that spanned through the entire lower leg created a tourniquet effect leading to compartment syndrome, vascular compromise, ischemia, and gangrene [6].

The lower leg becomes the most commonly affected by acute compartment syndrome as it is relatively limited compliance to accommodate the expansion secondary to hematoma or swelling. However, the atypical presentation of most cases may result in a delay in diagnosis or treatment, thereby increasing the likelihood of morbidity for the patient. The practice of the TBS, including their preference for no documentation, makes it difficult to recognize the clinical presentation and severity of the patient's injuries. Moreover, this could have been resolved conservatively by limb elevation, release of the tight splints, or by fasciotomy. Therefore, it is crucial to be mindful of compartment syndrome as early clinical suspicion is the key to reduce morbidity and mortality [6,13].

In most severe injury or multiple trauma cases, systemic inflammatory response syndrome (SIRS) commonly occurs within 30 min after a major injury, and it is an inflammatory response to blood loss and tissue damage rather than infection. SIRS results from the release of mediators, which can cause fever, toxemia, and sometimes organ failure [14]. Four SIRS criteria were defined as tachycardia (heart rate > 90 beats/min), tachypnea (respiratory rate > 20 breaths/min), fever or hypothermia (temperature > 38 or < 36 °C), and leukocytosis, leukopenia, or bandemia (white blood cells >1200/mm^3^, <4000/mm^3^ or bandemia ≥10%). SIRS diagnosed as if two or more of these criteria were established [15]. SIRS was observed in this patient based on the tachycardia, tachypnea, fever, and leucocytosis before presented to our hospital. Adequate resuscitation was necessary for the patient to be fit for anesthesia and required surgical procedure.

The radiograph of the patient showed gas in the soft tissue around the lower leg suggestive of gas gangrene. Mbada et al. reported that extremity gangrene (19.7%) was the second most commonly observed complication after malunion/nonunion (36%) following treatment of the TBS [3]. Gas-forming soft tissue infections may be caused by various organisms that produce varying sequelae and disease processes. Clostridial gas gangrene is a rapidly progressive, life-threatening infection resulting in skeletal muscle necrosis, which usually occurs after muscle injury and contamination. Facultative organisms *(Escherichia coli, Klebsiella, various streptococci*) and anaerobic bacteria (*Bacteroides, Peptostreptococcus*) have also been isolated from soft-tissue gas infections. Typically, these nonclostridial infections are more gradual in onset and progression, and systemic manifestations are milder. Deep cultures and bacterial biopsy are sometimes necessary to make a diagnosis [16]. However, in this case, financial reason was the primary concern why the biopsy could not be performed.

The primary goal of the treatment was to prevent further ascending infection and toxemia, which was achieved by amputation. The necrotic part is already extended up to the knee joint, including the lower thigh. The absence of popliteal pulse also strengthens the indication for the orthopedic surgeon to performs above-knee amputation. Prosthetic fitting may compensate for the loss of body structures and functions of the affected limbs [17]. However, a prosthetic limb could not be designed as lack of prosthetic services in Papua.

In presenting this neglected case, the authors propose showing progressive limb-threatening infection emphasizing the importance of early recognition of compartment syndrome, adequate resuscitation or stabilization, and prompt and aggressive treatment. Although the mortality rate of gas gangrene varies in the literature, it is clear that aggressive infection warrants surgical treatment, especially when accompanied by systemic manifestation [16]. Meanwhile, preventive measures should be advocated through public education and enlightenment about the effects of harmful practices of TBS. The underlying factor in the progress of this disease is the ignorance of the traditional bone setters and their inability to recognize the early problems associated with tight splints. Therefore, integrated training should also be provided for TBS on the basics of orthopedic care service. Government subsidy of hospital treatment costs and expansion of the National Health Insurance to cover fracture treatment procedures need to be applied to decrease morbidity and mortality in the near future [10].

## 4 Conclusion

TBS's gangrene is one of the devastating complications arising from the practice of traditional bone setters. Above-knee amputation is done as a life-saving procedure. The loss of the limb results in a lifetime disability, impacting the patient and family significantly. However, the public has a lot of confidence and belief in the ability of bone setters despite the complications. Therefore, this case is reported to highlight the menace of TBS activities and the danger inherent in their practice. People who are misguided by false beliefs should be educated by public enlightenment. Appropriate legislation has to be supplemented by the government to integrate traditional bone settings with the new orthopedic care service.

## Sources of funding

This study was funded independently.

## Ethical approval

We have conducted an ethical approval to the ethical committee of Jhon Piet Wanane General Hospital. The study was performed according to the rules of anonymity.

## Consent

Written informed consent was obtained from the patient for publication of this case report and accompanying images. A copy of the written consent is available for review by the Editor-in-Chief of this journal on request.

## Authors contribution

**Lu Jordy Luhur:** Conceptualization, Software, Data curation, Formal analysis, Investigation, Validation, Writing - Original Draft, Writing - Review & Editing, Visualization. **I Putu Gede Dharmawan:** Conceptualization, Supervision, Formal analysis, Supervision, Validation, Resources. **I Gusti Lanang Ngurah Agung Artha Wiguna:** Conceptualization, Supervision, Formal analysis, Validation.

## Registration of research studies

1. Name of the registry: A Catastrophic Presentation of Dead Limb Secondary to Traditional Bone Setter's Treatment in Sorong, West Papua: A Case Report.
2. Unique identifying number or registration ID: researchregistry6847
3. Hyperlink: https://www.researchregistry.com/browse-the-registry#home/registrationdetails/60a8f17d545379001e739820/

## Guarantor

Lu Jordy Luhur.

## Provenance and peer review

Not commissioned, externally peer-reviewed.

## Declaration of competing interest

There are no conflicts of interest.
